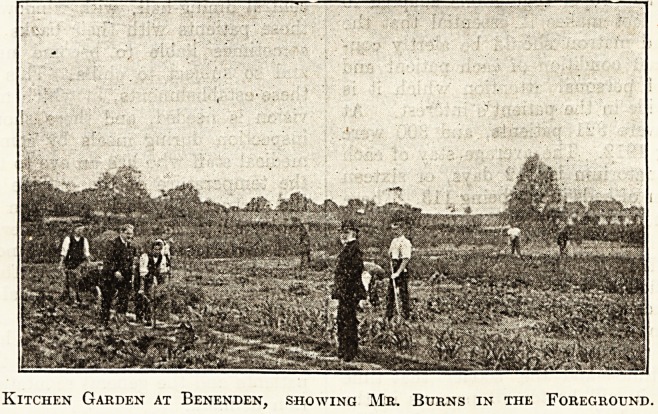# Reports on Hospitals of the United Kingdom

**Published:** 1914-04-04

**Authors:** Henry Burdett


### REPORTS ON

### Hospitals of the United Kingdom

By SIR HENRY BURDETT, K.C.B., K.C.V.O.

SERIES III.

### BENENDEN SANATORIUM, KENT.

This sanatorium has been established by the "National Association for the Establishment and Maintenance of Sanatoria for Workers suffering from Tuberculosis." The Association commenced its work in 1905. The Benenden Sanatorium contains 113 beds, of which ninety-four are maintained by Friendly Societies, Borough Councils, King Edward's Hospital Fund, the Hospital Saturday Fund, and the Orsett Joint Hospital Board. The site at Benenden is excellent, though exposed, situated as it is on high ground, commanding an extensive vista of fine country. The administration building occupies the centre, and there are pavilions of two storeys for patients in connection with, but on either side of, it. In addition, there are two other pavilions, called respectively the Post Office and Countess Cadogan pavilions. The cubicles in these pavilions are two-bedded; they have an abundance of sunlight and air, and should constitute not unpleasant accommodation for tuberculous cases. With the exception of a marquee accommodating ten patients, none of the wards, or cubicles, contains more than two beds each. There are forty of these double cubicles and twenty-three single ones.

Kitchen Garden at Benenden, showing Mr. Burns in the Foreground.

### An Ideal Shelter.

We were much struck with the apparent popularity among the patients of a large tent which contained some twenty patients, who seemed to be well contented and happy in their quarters. Tuberculosis is a disease which has a world-wide reputation for bringing cheerfulness with it to the patient. A sanatorium is not necessarily built for comfort, but for health; and as health conditions are mainly isolation in cubicles with the free circulation of fresh air, involving practically continuous residence in the open air, the patients, when they get accustomed to the treatment, find it hard indeed to return to an ordinary dwelling-house as a place of residence. The house resident's first impression of a sanatorium is not a favourable one for these reasons, but we found in the course of our inspections that there is a growing habit among the resident members of hospital staffs of both sexes to sleep in the open air wherever the opportunity is afforded.

In the grounds is an excellent smoking shed, designed by Mr. A. W. West, which is one of the very best erections of the kind we have ever seen. It contained several Lloyd Smith chairs, which are constructed so as to allow a convalescent to be moved about the garden and back to the ward. These chairs, from the advantages of their construction and durability, are to be found in increasing numbers in sanatoria. They are described and illustrated on page 19 of this issue.

### General Condition of the Institution.

The buildings, which are new, present, on the whole, a smart appearance, and are well kept, the pavilions containing two-bedded cubicles being especially attractive.

The sanatorium is constructed with long corridors, and has sunny day- and bed-rooms with a southern aspect. The site is extensive, and is admirably adapted to its purpose. Rest and exercise in the open air, including work and walking exercise, are prescribed and carefully regulated for each patient. We have little doubt that the work, which includes gardening, work on the farm, carpentry, and other branches within the compass of the patient, is an attractive feature of this institution.

The sanitary arrangements at this sanatorium include both water and earth closets. The earth closets were in good order and well kept; the water closets which we saw were not well kept, and it seemed to us that all of them ought to be inspected and thoroughly cleansed at least once in every twenty-four hours.

### Equipment and Staff.

We are of the opinion that, in view of the experience gained in the work of sanatoria during the last few years, it is desirable that some authoritative statistics, based upon the actual working and administration of existing sanatoria, should be published, giving particulars of the actual number and character of the staff—medical, nursing, and domestic—which sanatoria should employ in proportion to the number of patients admitted for treatment. The patients are rightly encouraged to be up and about under proper regulations adapted to each case. This fact makes it essential that the superintendent and matron should be alertly conscious of the actual condition of each patient and of the amount of personal attention which it is necessary to provide in the patient's interest. At Benenden there were 321 patients, and 300 were discharged during 1912. The average stay of each patient at this sanatorium is 112 days, or sixteen weeks, the number of beds in use being 113. There are three resident medical officers, a matron, and eight nurses.

We should like to know whether eight nurses is a sufficient staff, under all circumstances, to give adequate attention to the patients in 113 beds, when, on an average, some two-fifths to one-half of the patients are acutely ill. We look forward to the time—and the sooner that time arrives the better it will be for all persons in the first stage of tuberculosis—when a sanatorium like that at Benenden will confine its work entirely to the admission of these first-stage patients only. Until that time arrives a very grave responsibility attaches to everyone who is responsible for sending a case of tuberculosis in the first stage into a sanatorium where patients in the second and third and final stages of the disease are under treatment. The "Rules for Consumptives" at this sanatorium are intelligently drawn up, and contain useful information, simply stated and convincingly put.

### Results since 1907.

A careful attempt is made to trace every patient who has been under treatment at this sanatorium. Of fifty-four patients discharged in 1907, fourteen are still in full work, three have been re-admitted, and twenty-two are dead. In 1908, 154 patients were discharged, and of these twenty-five are still in full work, one on partial work, three are working, sixteen have been re-admitted, and sixty-six are dead. Of 234 discharged in 1909, thirty-nine are still on full work, six on partial work, fifteen have been re-admitted, four are not at work, and seventy-nine are dead. In 1910, those discharged numbered 265, of whom fifty-five are still on full work, five on partial work, twenty-one were re-admitted, ten are not at work, and ninety-four are dead. Out of 281 discharged in 1911, ninety-three are still on full work, fifteen on partial work, eight were re-admitted, eighteen are not at work, and fifty are dead. It must be noted that the difference between the total number discharged and the number accounted for represents the discharged patients who are still untraced.

### Sanatoria Dining-Halls.

The dining-hall at Benenden is a well-constructed, well-equipped feature of the buildings, it presents an attractive appearance, and in making the following remarks we wish it to be understood that they are made generally and not specially in relation to the sanatorium at Benenden. From statements made to us by patients who have been inmates of sanatoria we gather that in a central dining-hall, with windows open all round, those patients with their backs towards them are sometimes liable to become uncomfortably cold and so subject to chills. This is a direction, in these establishments, in which much closer supervision is needed, and there should be continuous inspection during meals by some member of the medical staff who has an eye to the weather and to the temperature of the outside air, as well as to the actual physical condition of the patients subjected to the risks just referred to. Adequate and perfect ventilation should not mean perpetual draughts, and the regulation of the windows and the admission of air, during meal-times in central dining-rooms in sanatoria, is an administrative detail which does not appear to have had the attention given to it in the past that the interests of the patients and the general reputation of each institution demand.

It does not seem to have been sufficiently apprehended that it is one thing to place a patient, when in bed, in a pavilion with all the windows open, and quite another to place the same patient in a dining-hall under similar conditions. The effect of open windows all round and of cross-currents throughout the dining-hall cannot be beneficial to the patient. It does not constitute thorough ventilation in the proper meaning of the words, and such arrangements do in fact often bring unnecessary discomfort and even worse to many patients, whilst it is impossible, in all weathers and under varying degrees of temperature, under such conditions to serve a hot meal comfortably, or to secure that each patient's portion shall be served hot. By all means let every patient at the same time be well protected from the evils referred to in the course of this criticism of the conditions which prevail in the dining-halls of so many sanatoria.

## Figures and Tables

**Figure f1:**